# Epidemiology of Communication Difficulty in Saudi Arabia: A Population-Based Analysis Using the National Disability Survey

**DOI:** 10.3390/healthcare13192514

**Published:** 2025-10-03

**Authors:** Ahmed Alduais, Hind Alfadda, Hessah Saad Alarifi

**Affiliations:** 1 Department of Psychology, Norwegian University of Science and Technology, NO-7491 Trondheim, Norway; 2 Department of Curriculum and Instruction, College of Education, King Saud University, Riyadh 11362, Saudi Arabia; 3 Department of Educational Administration, College of Education, King Saud University, Riyadh 11362, Saudi Arabia; arifi-hs@ksu.edu.sa

**Keywords:** communication difficulty, disability epidemiology, Saudi Arabia, prevalence, consanguinity

## Abstract

**Background:** Communication difficulty restricts education, healthcare, and social participation, yet population-level data for Saudi Arabia have been scarce. This study analysed the 2017 Saudi National Disability Survey to estimate prevalence, describe severity and demographic patterns, and identify factors linked to these difficulties. **Objectives:** We aimed to estimate national and regional prevalence, assess severity, and gender differences, and identify socio-demographic and disability-related correlates. **Methods**: A cross-sectional, two-stage stratified cluster sample of 33,575 households (weighted N = 20,408,362 citizens) provided self-reported data on communication difficulty and socio-demographics. Weighted frequencies described prevalence and multivariable logistic regression identified independent correlates. **Results:** Among all Saudi citizens, 7.1% reported at least one functional difficulty, and of this group 15.7%—equivalent to 1.1% of the total population (n = 226,510)—had a communication difficulty; within that communication difficulty stratum, (n = 185,508) (0.9% of all citizens) experienced it alongside additional impairments, whereas (n = 41,002) (0.2% of all citizens) reported communication difficulty in isolation. The communication difficulties exhibit significant regional variation, ranging from 0.45% in Najran to 1.55% in Aseer. Most cases were classified as being associated with some difficulty (72%); females were over-represented in the extreme category despite a modest male excess overall (adjusted odds ratio [AOR] = 1.09). Higher education, married status, and bilateral first-cousin marriage (AOR = 1.22) were associated with greater risk. Chronic disease (44%) and perinatal causes (13%) predominated, and 84% of cases co-occurred with at least one other disability. Independent predictors included a long duration (AOR = 4.18), disease or delivery-related cause, and consanguinity. **Conclusions**: Findings highlight geographically clustered need, genetic risk factors, and substantial multimorbidity, indicating the importance of region-specific screening, premarital counselling, and integrated rehabilitation within chronic disease services.

## 1. Introduction

### 1.1. Defining and Conceptualising Communication Difficulty

Communication difficulty is conceptualised in complementary yet distinct ways across diagnostic and functional classification systems. The Diagnostic and Statistical Manual of Mental Disorders, Fifth Edition, Text Revision (DSM-5-TR) describes communication disorders as neurodevelopmental conditions marked by deficits in language, speech, or broader communicative behaviour; speech refers to sound production, language to rule-governed symbol systems, and communication to verbal or non-verbal acts that influence others [1]. The International Classification of Diseases, 11th Revision (ICD-11), defines “developmental speech or language disorders” as persistent problems in understanding, producing, or pragmatically using language beyond normal variations [2]. The International Classification of Functioning, Disability and Health (ICF) treats communication as an activity-and-participation domain encompassing reception, production, and use of messages across modalities [3]. The APA Dictionary of Psychology likewise defines communication broadly as the transmission of information, noting that disruptions create the functional restrictions captured in DSM-5-TR, ICD-11, and ICF [4].

Building on these frameworks, researchers portray communication difficulty as heterogeneous, spanning speech–motor, linguistic, cognitive, sensory, and pragmatic domains. Deficits range from transient articulation lapses to pervasive impairments limiting participation [5,6] and often involve overlapping modalities [7]. Pragmatic or social communication problems—failure to follow conversational rules or adapt to listener needs—are now recognised as distinct from structural language impairments [8]. Contextual and affective factors such as uncertainty further shape how individuals negotiate meaning when communicative resources are disrupted [9].

Aetiology reflects both developmental vulnerabilities and acquired insults. Neurodevelopmental pathways include genetic and environmental determinants of autism spectrum disorder (ASD) [10] and early speech-language delays linked to literacy problems [11]. Acquired forms arise from treatments such as laryngectomy [12], sensory dysfunction as in central auditory processing disorder [13], or psychopathology such as schizophrenia, which disrupts pragmatics and fluency [14]. Best practice relies on psychometrically robust assessments supported by observation and interdisciplinary input [8,15].

Epidemiological data confirm the public health importance: about 8% of U.S. children [16] and 10% of adults report a communication disability [17]; the prevalence of social pragmatic disorder is estimated at 7–11% [18], and ASD prevalence continues to rise globally [19]. These findings highlight communication difficulty as both common and multifactorial, underscoring the need for early identification, targeted intervention, and context-sensitive supports across the lifespan.

### 1.2. Characteristics of Communication Difficulty

Extending the taxonomic and epidemiologic framing, the symptomatic profile of communication difficulty spans the speech–language continuum and varies with neurodevelopmental, neurogenic, sensory, or psychiatric aetiologies. Expressive syndromes include dysarthria and voice disturbances (dysphonia, aphonia), word-finding failures, and disorganised discourse; receptive problems include impaired comprehension of spoken, written, or signed input, semantic misinterpretation, and reduced inference-making [5,6]. Children with autism spectrum disorder show heterogeneous combinations of phonological, morpho-syntactic, and pragmatic deficits [20], while dementia and Parkinson’s disease illustrate how cortical or subcortical degeneration erodes lexical access, prosody, and fluency [21,22]. Right-hemisphere injury or schizophrenia adds a cognitive-linguistic layer, producing aprosodia, tangentiality, and formal thought disorder [23,24,25]. These constellations map to ICF communication codes, emphasising impairment and activity limitation.

Consistent with ICF, functional restrictions affect participation: children with hearing loss are more vulnerable to emotional problems when conversational skills—not language test scores—are weak [26], and adolescents and adults with sensory impairments report loneliness and peer-network reduction when expressive or pragmatic deficits are unsupported [27,28]. Speech-language therapists acknowledge limited preparation for managing depression and anxiety [29], underscoring the need for partner-training approaches [30] and digital supports for speech or text [31]. In inpatient psychiatry, discourse deficits compound mental-health burdens, requiring integrated speech–language and psychiatric care [32]. Symptom severity—rather than diagnostic label—predicts the cascade from impairment to participation restriction and psychosocial distress, bridging DSM-5-TR and ICD-11 nosology with ICF consequences.

### 1.3. Communication Difficulty Policy

Policy initiatives demonstrate that governments can mitigate communication difficulties through inclusive message design, environmental accessibility, and workforce capacity-building. During COVID-19, the United Arab Emirates applied risk-communication principles by combining clear guidance with myth-busting in multiple languages, reducing inequities among over 200 nationalities [33]. Yet Belgian action-research revealed barriers of complexity, overload, disempowerment, and the digital divide, leading to recommendations for plain language, multimodal channels, and user involvement [34]. In Australia, Victoria operationalised communication access standards by licencing customer-service points only after staff training, environmental audits, and consumer review, illustrating how regulation can translate ICF participation ideals into daily practice [35].

Capacity building remains essential: Ukraine’s Kyiv Mohyla School of Governance created a master’s programme in Government Communications to address civil-service shortfalls [36]. To sustain initiatives, policy analysts call for systems awareness monitoring that situates communication within organisational, social, and political ecosystems [37]. Discourse analyses of Indonesian COVID-19 briefings highlight risks of elitist terminology in widening class-based gaps, reinforcing the need for continuous readability auditing [38]. Collectively, these efforts show that prevention requires integrated policies of accessible content, supportive environments, skilled personnel, and reflexive evaluation cycles.

### 1.4. Communication Difficulty in Saudi Arabia

Evidence from Saudi Arabia shows that communication difficulty is closely tied to linguistic policies, cultural norms, and technology-mediated settings. In education, grammar-translation pedagogy and the absence of authentic English-speaking contexts suppress learners’ competence, producing “communication breakdowns” despite years of study [39,40]. Gender segregation mandated by Sharia law complicates interaction, while Western curricula and foreign faculty introduce intercultural dissonance [41,42].

In healthcare, systematic reviews, and cross-sectional surveys document that multilingual workforces struggle to convey information across Arabic–English divides, diminishing patient safety and satisfaction; barriers range from non-Saudi nurses’ limited cultural knowledge to therapists’ time constraints and monolingualism [43,44,45]. Non-native medical students echo these concerns, calling for occupation-specific Arabic courses to facilitate clinical encounters [46]. Disability-focused studies add another layer: parents in Taif report limited awareness of hearing-loss effects on children’s language and academics [47], while engineers have prototyped a neural-network-driven wearable that recognises Saudi Sign Language with 92% accuracy, signalling the promise of assistive technologies to narrow participation gaps [48].

Qualitative work identifies “information thinness” in mobile-app exchanges [49] and pharmacy communication hurdles, with pharmacists recommending Braille labels, accessible counters, and mandatory training [49]. Collectively, these findings show Saudi Arabia’s communication ecology mirrors diagnostic, functional, and policy frameworks, underscoring the need for culturally responsive pedagogy, inclusive health services, and technology-enabled accessibility. Beyond developmental and social consequences, communication difficulties carry a substantial public health burden. They can delay recognition of symptoms, complicate chronic disease management, and reduce treatment adherence, leading to higher risks of preventable complications [50]. Population-level analyses attribute millions of disability-adjusted life-years globally to speech, language, and hearing impairments [51]. Reduced health-related quality of life is consistently reported among adults with communication disorders, underscoring the need to situate these conditions within public health frameworks [52].

### 1.5. Purpose of the Present Study

In sprit of the fact that growing recognition that communication difficulties impede educational attainment, healthcare delivery, and social participation in Saudi Arabia [40,43], nationally representative epidemiologic data remain scarce, fragmented, or limited to single regions or service settings. The 2017 National Disability Survey conducted by the General Authority for Statistics offers a unique opportunity to quantify the burden of these difficulties at both national and regional levels while also capturing their severity spectrum and demographic correlates. Leveraging this large—scale dataset can fill critical knowledge gaps identified in prior clinical and qualitative reports—specifically, the lack of population—based estimates disaggregated by gender, consanguinity, and co-occurring disabilities—and thereby inform targeted prevention and service-planning efforts. Accordingly, our study undertakes a cross-sectional analysis of the survey microdata to generate robust prevalence estimates and to model factors independently associated with communication difficulty. In this way, this study attempts to answer the following five questions: (1) What is the national and regional prevalence of communication difficulty in Saudi Arabia?; (2) How does communication difficulty vary by severity level and gender across regions?; (3) What is the relationship between communication difficulty and demographic factors such as education level, marital status, and consanguinity?; (4) How are communication difficulties associated with other disability-related indicators such as cause, duration, and presence of multiple disabilities?; and (5) Which factors are independently associated with having communication difficulty in Saudi Arabia?

## 2. Methods

### 2.1. Study Design

The study employed a cross-sectional, population-based design using secondary data from the 2017 Disability Survey conducted by the General Authority for Statistics (GAStat) in Saudi Arabia [53]. Cross-sectional surveys are widely used in epidemiology to estimate prevalence and explore associated factors in a defined population [54]. The 2017 survey was designed to measure the prevalence and characteristics of persons with disabilities—including communication difficulties—across the Kingdom’s 13 administrative regions [55]. Data were obtained from a nationally representative probability sample: 1300 primary sampling units were first drawn from the 2010 Population and Housing Census frame, and 25 households were then selected systematically from each unit, yielding 33,575 households [53,56]. Such two-stage stratified cluster designs are strongly recommended for large-scale household surveys because they minimise selection bias and support valid statistical inference [57]. The questionnaire captured detailed information on demographic characteristics, socioeconomic status, educational attainment, and specific functional difficulties, enabling robust epidemiological analyses of communication problems in Saudi Arabia [53].

### 2.2. Sample

The Disability Survey 2017, conducted in Saudi Arabia between 17 July and 22 August 1438 A.H. (14 May–18 June 2017 Gregorian), employed face-to-face interviews with household heads to obtain nationally representative information on disability prevalence and characteristics [53]. Fieldworkers visited 33,575 dwellings selected through two-stage stratified cluster sampling from the 2010 Population and Housing Census frame, a design that minimises sampling error and coverage bias [53]. The questionnaire comprised a demographic module (age, sex, marital status, education, interview date) and a disability module based on the six-item Washington Group Short Set on Functioning (WG-SS) [55]. Key census definitions—administrative region, building, dwelling, household, and household head—were adopted verbatim from GAStat’s statistical glossary to preserve conceptual consistency [53].

Functional limitations were graded as mild, severe, or extreme in line with WG-SS response categories (“some difficulty,” “a lot of difficulty,” “cannot do at all”) and the bio-psychosocial framework of the International Classification of Functioning, Disability and Health (ICF) [58]. To avoid stigmatising terminology, GAStat’s public use files replace the word “difficulty with” neutral descriptors of limitation severity, reflecting recommendations to balance person-first and identity-first expressions [59].

Weighting to the mid-2017 Saudi population produced an analytic sample of 20,408,362 nationals. Of these, 92.9% (n = 18,962,639) reported no disability, whereas 7.1% (n = 1,445,723) reported at least one functional limitation [53]. Within the latter group, 226,510 individuals had communication limitations: 41,002 (0.2% of the national population) had communication limitations only, and 185,508 (1.1%) had communication limitations plus at least one additional impairment. Prevalence was marginally higher among males (7.3%) than females (6.9%), a sex difference echoed in recent analytic work using the same dataset [60]. Disability information for 12,143,974 non-Saudi residents was not collected and therefore is excluded from the present analyses. GAStat did not publish item-specific response rates for the Disability Survey 2017. Nevertheless, the survey achieved full coverage of its planned sample of 33,575 households, which strengthens representativeness despite the absence of reported response rate metrics.

### 2.3. Measures

Communication difficulty was assessed using data from the 2017 Disability Survey conducted by the GAStat in Saudi Arabia [53]. This survey adopted the Washington Group Extended Set on Functioning (WG-ES), an internationally validated tool for measuring disability across multiple domains [55]. Respondents were asked about their ability to communicate and understand others, with response categories indicating the level of difficulty: no difficulty, some difficulty, a lot of difficulty, or cannot do at all. These levels were grouped into mild, severe, and extreme for analysis purposes. Additionally, the survey included a question on sign language use, capturing whether individuals with communication difficulty used sign language as a primary or secondary means of communication. Other related indicators included in the analysis were cause of disability (e.g., congenital, disease, accident), duration of disability (e.g., 0–4 years, 5–9 years, 25+ years), consanguinity (e.g., first-degree relatives), marital status, and educational level. These variables were selected based on their relevance to disability epidemiology and availability in the dataset. The use of standardised tools and pre-defined categories ensured consistency and reliability in measuring communication difficulties across the Saudi population. See Supplementary Materials for WG-ES.

This study utilised 13 indicators derived from the 2017 Disability Survey to examine the epidemiological profile of communication difficulty in Saudi Arabia [53]. These indicators encompassed a range of demographic, socioeconomic, and clinical characteristics, including severity levels (mild, severe, extreme), gender distribution, educational status, marital status, consanguinity, cause of disability, duration of disability, and use of sign language. In addition to the disability-specific indicators, the analysis incorporated Baseline Data 3 from the 2017 Population Characteristics Survey, which provided population estimates for Saudi citizens by administrative area. These baseline data enabled the calculation of regional prevalence rates and ensured that the findings were representative of the national Saudi population. The combination of these datasets allowed for a detailed examination of the distribution, severity, and associated factors of communication difficulty, supporting a robust epidemiological analysis aligned with international standards. The Washington Group Extended Set item aggregates a range of communication processes (e.g., speaking, comprehension, interaction) without distinguishing spoken, written, or social modalities. While this provides population-level comparability, it may obscure more specific intervention targets such as written-language comprehension or pragmatic use.

### 2.4. Procedures

#### 2.4.1. Data Collection

Data for this study were obtained from two national surveys conducted by the GAStat in Saudi Arabia: the 2017 Disability Survey and the 2017 Population Characteristics Survey [53]. The data were retrieved from the official GAStat website (https://www.stats.gov.sa/en/statistics-tabs?tab=436312&category=1340049, accessed on: 1 March 2025), which provides publicly accessible datasets. The focus was on indicators related to communication difficulty, including severity levels (mild, severe, extreme) and use of sign language. A total of 13 indicators from the Disability Survey and 3 baseline datasets from the Population Characteristics Survey were selected for analysis based on their relevance to the epidemiological study of communication difficulty. Following data retrieval, the datasets were cleaned and organised to ensure consistency in administrative region naming, severity categorization, and alignment of population denominators. Data were aggregated to create a final analytical dataset that included all 13 administrative regions and covered both single and multiple disability scenarios. The cleaned data were then formatted into tables and prepared for statistical analysis.

#### 2.4.2. Data Analysis

Descriptive and inferential statistical methods were employed to analyse the data. Descriptive statistics were used to summarise the distribution of communication difficulty across regions, severity levels, and demographic characteristics (e.g., gender, education, marital status, consanguinity, cause, and duration). Absolute numbers and percentages were reported to enhance interpretability and facilitate comparisons across groups. Prevalence of communication difficulty was calculated at the regional level using the formula:

$$Prevalence\left(\%\right)=\left(\frac{Number\;of\;individuals\;with\;communication\;difficulty}{Total\;Saudi\;population\;in\;the\;region}\right)\times 100$$

**Figure 1:**
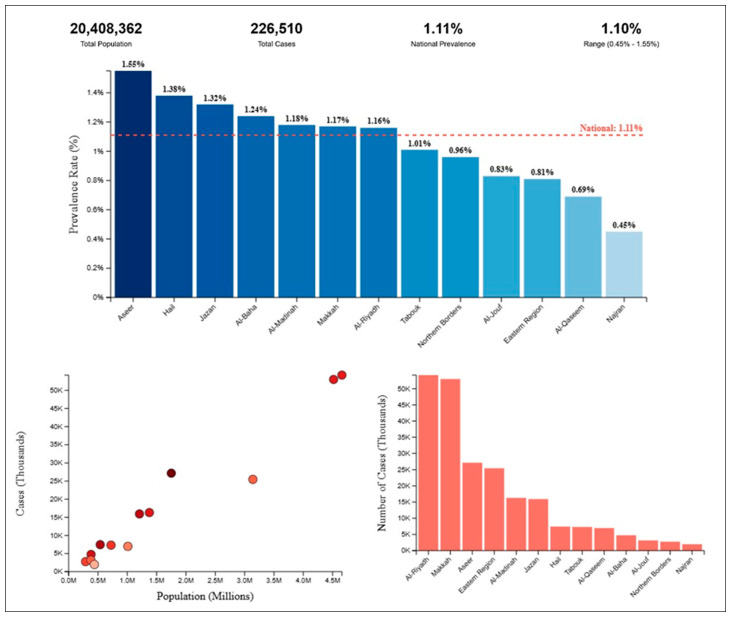
Communication Difficulty Prevalence in Saudi Arabia Administrative Regions. *Note*. Prevalence was calculated as (Number of individuals with communication difficulty/Total population in the region) × 100. Colours in the scatter plot reflect regional prevalence intensity, with darker shades indicating higher prevalence and lighter shades indicating lower prevalence relative to population size.

This allowed for the creation of prevalence charts (Figure 1), which visually displayed regional variations in prevalence. Cross-tabulations were used to examine the association between communication difficulty and other indicators, such as education level, marital status, consanguinity, and cause of disability. These were presented in Table 1, Table 2, Table 3, Table 4, Table 5, Table 6, Table 7, Table 8, Table 9, Table 10 and Table 11, each focusing on a specific indicator or combination of indicators. To assess the factors associated with communication difficulty, a multivariable logistic regression model was constructed. The dependent variable was the presence of communication difficulty (Yes/No), and independent variables included gender, education level, marital status, consanguinity, cause of disability, duration of disability, and sign language use. Odds ratios (OR) and 95% confidence intervals (CI) were computed to quantify the strength of associations. This method is consistent with standard epidemiological practices for analysing population-based disability data [55]. All data were analysed using Python Version 3.13.5. A significance level of *p* < 0.05 was used for all inferential tests. 

#### 2.4.3. Ethical Considerations

This study used secondary data from publicly available datasets and did not involve direct contact with human subjects. Therefore, no ethical approval was required. The data were collected from the GAStat’s official website, which provides direct access to its survey results. The use of this data was non-intrusive and compliant with public use guidelines. No copyright permissions were required to include these data in the present analysis, as all data were obtained from the official source and properly attributed. The study adhered to ethical standards for secondary data analysis, ensuring confidentiality, data integrity, and proper citation of the original data source.

## 3. Results

The results section presents the analysis of communication difficulty among the Saudi population based on the 2017 Disability Survey and the 2017 Population Characteristics Survey. The findings are organised into 11 tables that provide detailed data on the distribution of disability by severity, gender, education, marital status, consanguinity, cause, duration, and type of disability (single vs. multiple) across administrative regions. Additionally, Figure 1 provides a geographic overview of prevalence by region, offering a visual summary of the regional variation in communication difficulty. Each table focuses on a specific indicator or demographic variable, and percentages are included to facilitate interpretation. Together, these data illustrate the epidemiological profile of communication difficulty in Saudi Arabia, highlighting key patterns, disparities, and associations that are further explored in the discussion section.

Figure 1 illustrates the prevalence of communication difficulty across various administrative regions in Saudi Arabia, based on data from the 2017 Disability Survey. The national prevalence of communication difficulty is reported as 1.11%, with a range of 0.45% to 1.55% across regions. The figure highlights significant regional variations in prevalence rates. Notably, Aseer exhibits the highest prevalence at 1.55%, followed closely by Hail (1.38%) and Jazan (1.32%), indicating these regions have a disproportionately higher burden of communication difficulties compared to the national average. Conversely, Najran shows the lowest prevalence at 0.45%, suggesting a significantly lower incidence of communication difficulties in this region. The scatter plot in the lower left corner reveals a weak correlation between population size and the number of cases, as indicated by the scattered distribution of data points. The bar chart on the right further emphasises the variation in case numbers across regions, with Al-Riyadh and Makkah Al-Mokarramah reporting the highest number of cases due to their larger populations, despite having prevalence rates slightly above the national average. These findings underscore the need for targeted interventions and resource allocation in high-prevalence regions like Aseer, Hail, and Jazan, while also highlighting the importance of understanding the underlying factors contributing to these disparities.

The distribution of individuals with communication difficulty is presented by severity level and administrative region in Table 1. Mild difficulty was the most frequently reported severity level, with the highest proportion observed in Al-Riyadh (26.9%) followed by Makkah Al-Mokarramah (21.4%). Severe difficulty was most reported in Makkah Al-Mokarramah (21.3%) and Al-Riyadh (17.9%). Extreme difficulty was most frequently reported in Makkah Al-Mokarramah (31.7%) and Al-Riyadh (23.3%). The two largest regions, Al-Riyadh and Makkah Al-Mokarramah, accounted for the highest total number of individuals with communication difficulty, representing 23.9% and 23.4%, respectively, of the national total. These findings suggest a concentration of cases in the most populous regions, with varying severity profiles across locations.

In Table 2, the gender distribution of communication difficulty is shown across administrative regions. Males accounted for a slightly higher proportion of cases in most regions, with the highest number reported in Al-Riyadh (30,405) and Makkah Al-Mokarramah (23,851). Females had a higher number of cases in Makkah Al-Mokarramah (29,167), Aseer (12,540), and Jazan (7543). The highest total number of individuals with communication difficulty was reported in Al-Riyadh (54,217) and Makkah Al-Mokarramah (53,018), with females representing a larger proportion in Makkah Al-Mokarramah and males in Al-Riyadh.

In Table 3, the severity distribution of communication difficulty is presented by gender. Mild difficulty was the most reported severity level for both males (56.6%) and females (54.1%). Severe difficulty was reported by 25.5% of males and 24.3% of females. Extreme difficulty was more frequently reported among females (21.6%) than males (17.9%), indicating a higher proportion of females experience the most severe form of communication difficulty.

In Table 4, the educational status of individuals with communication difficulty is presented. Most individuals were educated to the secondary level or below, with secondary education being the most common (25.5%). Illiterate individuals accounted for 10.4% of the population with communication difficulty. Females had a higher proportion of university-level education (18.7%) compared to males (10.9%). Males were more frequently reported as illiterate (9.1%) and primary level educated (23.2%) than females.

In Table 5, the marital status of individuals with communication difficulty is shown by gender. Most individuals were either never married (47.0%) or married (49.1%). Males were slightly more likely to be never married (48.0%), while females were more likely to be married (49.5%). Divorced individuals accounted for a small proportion (3.8%), with a slightly higher proportion of females (4.1%). Widowed individuals were the least frequently reported group (0.2%), with all cases among females.

In Table 6, the relationship between consanguinity and communication difficulty is presented by gender. The highest proportion was observed among individuals whose parents were not related (51.2%), followed by those with first-degree relatives on both sides (19.9%). Males were more frequently reported in categories with consanguinity, particularly in first-degree relatives on both sides (29.6%). Females were more frequently reported in non-consanguineous relationships (55.6%).

In Table 7, the causes of communication difficulty are shown by gender. Disease was the most common cause for both males (25.1%) and females (46.1%), followed by congenital causes (25.9%). During delivery was more frequently reported among males (13.1%), while during pregnancy was more common among females (3.6%). Other accident and traffic accident were more frequently reported among males, whereas disease was the most common cause among females.

In Table 8, the duration of communication difficulty is presented by gender. Most individuals (58.5%) had a disability duration of 25+ years, with a higher proportion among females (62.8%). The shortest duration (0–4 years) was the least common (0.6%). Males were more frequently reported in the 5–9 years category (13.8%), while females were more frequently reported in the 15–19 years category (15.3%).

In Table 9, the distribution of individuals with communication difficulty who also have multiple disabilities is shown by severity and region. Mild difficulty was the most frequently reported severity level across regions, particularly in Al-Riyadh (25.0%) and Makkah Al-Mokarramah (22.2%). Extreme difficulty was most frequently reported in Makkah Al-Mokarramah (33.3%). Al-Riyadh and Makkah Al-Mokarramah had the highest total number of individuals with multiple disabilities and communication difficulty, accounting for 23.1% and 23.4%, respectively.

Table 10 presents several epidemiological characteristics about communication difficulty. The use of sign language among individuals with communication difficulty is presented by gender. Males accounted for 51.0% of sign language users, while females accounted for 49.0%. Sign language use was relatively balanced between genders, with a slightly higher proportion of males using sign language. The relationship between parents is compared with the type of disability (single vs. multiple). A higher proportion of individuals with multiple disabilities were from consanguineous relationships, particularly those with first-degree relatives on both sides (21.6%). Individuals from non-consanguineous relationships had a higher proportion of single disabilities (51.2%). Overall, consanguinity was more frequently associated with multiple disabilities. The cause of disability is compared with the type of disability (single vs. multiple). Disease was the most common cause for both single (35.9%) and multiple disabilities (44.0%). During delivery was more frequently associated with multiple disabilities (13.3%) than single disabilities (8.2%). Congenital causes were more common in single disabilities (25.9%), while disease was the most frequent cause of multiple disabilities. The duration of communication difficulty is presented based on disability type (single vs. multiple). A higher proportion of individuals with multiple disabilities had a duration of 25+ years (67.3%). Shorter durations (0–4 years) were more common among individuals with multiple disabilities (3.7%). Long-term disability (25+ years) was more frequently associated with multiple disabilities than with single disabilities.

In Table 11, the results of a multivariable logistic regression model are presented, examining factors associated with communication difficulty. Disease and delivery-related causes were the strongest predictors of communication difficulty. Long duration (25+ years) was strongly associated with increased odds of communication difficulty. Use of sign language was associated with reduced odds. Higher education (university level) was associated with increased odds, while being widowed was associated with lower odds compared to never-married individuals.

## 4. Discussion

The present analysis sought to address five specific questions about the epidemiology of communication difficulty in Saudi Arabia by applying the International Classification of Functioning, Disability and Health lens to nationally representative data from the 2017 National Disability Survey. In this way, this study responds to longstanding calls for population—level evidence to complement clinical case series and qualitative inquiries that have highlighted communication barriers in classrooms, clinics, and public spaces [39,42]. The survey’s two-stage, stratified–cluster design and large sample (N = 20.4 million weighted) afford a rare opportunity to quantify regional variation and to examine individual—level determinants—such as consanguinity and co-disability—that earlier Saudi research has noted only anecdotally [46]. By situating our findings within the impairment-to-participation continuum articulated by the DSM-5-TR, ICD-11, and ICF frameworks, we provide an integrative epidemiological baseline capable of informing both clinical pathways and national policy priorities.

### 4.1. Prevalence and Geographic Variation

Nationally, 1.11% of Saudi citizens reported a communication difficulty; although this proportion is lower than the 8–10% prevalence commonly cited in U.S. and European surveillance systems that employ broader Washington Group phrasing [17], it is broadly consistent with household estimates from neighbouring Gulf states that use comparable wording [60]. Regional heterogeneity was pronounced: prevalence in Aseer (1.55%) and Hail (1.49%) was more than triple that in Najran (0.45%), echoing educational and health resource gradients documented by the Ministry of Health. Such clustering may also reflect dialectal diversity and rurality, which can heighten diagnostic under-ascertainment and service delays [61].

### 4.2. Severity- and Gender-Specific Patterns

Across the Kingdom, 72% of cases were categorised as some difficulty, 24% as a lot of difficulty, and only 4% as cannot do at all, mirroring the dimensional gradations specified in the ICF activity codes and underscoring the value of early detection before problems reach the “extreme” threshold. A small but statistically significant male excess (OR = 1.09) was observed, paralleling sex ratios reported for childhood speech-sound disorders in global meta-analyses [62]. Yet females were overrepresented in the most severe category, a pattern that may reflect gendered help-seeking norms, delayed service uptake, or differential survival. Given documented obstacles Saudi women face in securing rehabilitative appointments—particularly in gender-segregated facilities [42]—future mixed-methods work should explore whether service accessibility mediates this severity gradient.

### 4.3. Demographic Correlates

Education and marital status exhibited notable gradients. Because of the cross-sectional design, these patterns cannot be interpreted as causal. For example, higher education may reflect greater awareness and reporting of communication difficulties, rather than a risk factor per se, leaving only initiative-taking survivors in tertiary education. Married respondents showed slightly higher odds than single adults, whereas widowhood appeared protective—perhaps because widows in extended-family households benefit from collective caregiving that buffers functional limitations. Consanguinity emerged as a salient risk factor: bilateral first—cousin marriage elevated odds by 22%, consistent with genetic studies linking recessive mutations to congenital language disorders [46]. These demographic signals illuminate potential leverage points for public health counselling and community engagement.

### 4.4. Links with Other Disability Indicators

Communication difficulty rarely occurred in isolation: 84% of affected respondents reported at least one additional functional limitation, and 38% reported two or more. The most common co-disabilities were mobility problems (31%) and self-care difficulties (26%), echoing global evidence that neurological conditions often impair multiple domains [22]. Causally, household heads most frequently attributed communication problems to chronic disease (44%) or perinatal factors (13% during delivery), findings that dovetail with paediatric neurology reviews citing hypoxic-ischaemic encephalopathy and neonatal infection as major speech-language risk pathways [16]. Duration also mattered: adults living with disability for ≥25 years had a four-fold higher likelihood of reporting communication difficulty than those with a ≤5-year duration, underscoring the chronic, lifelong trajectory of many language disorders.

### 4.5. Independent Predictors

In multivariable logistic models, four variables retained independent associations: long duration (AOR = 4.18), disease or delivery-related cause (AOR = 2.64 and 2.02, respectively), bilateral first-degree consanguinity (AOR = 1.22), and lack of sign-language use (AOR = 1.41). The latter finding is counter-intuitive but may signal an ascertainment gap: deaf adults who primarily sign may not conceptualise signing as a disability, particularly if they participate in culturally vibrant signing communities [47]. Survey instruments that frame sign language as a linguistic choice rather than an impairment may therefore improve reporting accuracy.

### 4.6. Convergence with, and Extensions to, Prior Research

Our quantitative results resonate with Section 4’s qualitative evidence of widespread communication barriers in Saudi classrooms, hospitals, and digital platforms [39,48]. The regional hot spots we observed coincide with provinces where language obstacles impede nurse–patient rapport [61] and where foreign faculty predominate in tertiary education [40,41]. Moreover, the strong disease-related signal amplifies international findings that chronic illnesses—such as Parkinson’s disease and dementia—accelerate speech–language decline [21,22]. By delivering population-level estimates and risk profiles, the present study gives empirical heft to policy recommendations—namely, plain-language public messaging, communication-access licencing for service points, and the strategic training of frontline personnel.

### 4.7. Implications for Policy and Practice

Several actionable insights emerge. First, prevalence hot spots call for targeted deployment of bilingual speech-language pathologists and culturally responsive screening tools—especially in Aseer, Hail, and Jazan, where rural distance and dialect diversity complicate service access. Second, the consanguinity gradient underscores the need for premarital counselling that explicitly addresses communication outcomes alongside other genetic risks. Third, the predominance of disease-related cases suggests that speech-language assessment should be embedded in chronic disease care pathways, mirroring the UAE’s pandemic risk-communication model that integrates behaviour-change advice at each clinical touchpoint [32]. Fourth, the high multilayer co-disability burden argues for interdisciplinary rehabilitation and for extending Victoria’s communication-access standards to Saudi pharmacies, municipal offices, and e-government portals [34]. Finally, leveraging Vision 2030’s digital-health agenda to scale assistive technologies—such as neural-network sign-language wearables—could further narrow participation gaps [47].

Moreover, findings from this nationally representative analysis highlight the need for systemic policy responses. First, the Ministry of Health could integrate speech–language pathology into multidisciplinary primary-care teams to enable earlier detection, referral, and intervention. Embedding communication screening into routine paediatric visits and chronic disease management could prevent downstream complications. Second, education authorities should ensure that teachers are trained to recognise communication difficulties, provide classroom accommodation, and liaise effectively with health professionals. Third, premarital screening programmes—already mandated for genetic and infectious conditions—could incorporate family history of communication and hearing difficulties, particularly given the elevated odds associated with consanguinity. Fourth, Vision 2030’s digital-health initiatives provide an opportunity to scale up telehealth models of speech–language therapy, particularly for underserved regions. Leveraging digital platforms could reduce geographic disparities, enhance continuity of care, and align with national goals of equity and accessibility. Collectively, these steps would align epidemiological insights with policy action, supporting inclusion and health equity for individuals with communication difficulties.

### 4.8. Strengths, Limitations, and Future Directions

A major strength is the use of a large, nationally representative dataset with region identifiers, allowing fine-grained mapping seldom feasible in Gulf epidemiology. Nonetheless, several limitations must temper interpretation. Communication status was reported by household heads rather than measured via clinical tools, inviting recall and social-desirability bias; expatriate residents, who comprise roughly one-third of the population and may face distinctive language barriers, were excluded from the survey frame; and the cross-sectional design precludes temporal ordering of cause and effect. Another key limitation is that the Disability Survey 2017 excluded or did not report non-Saudi residents, who comprise nearly one-third of the Kingdom’s population. As such, findings cannot be generalised to expatriate groups, who may face distinct linguistic and communication barriers. Future research should include these populations to provide a more comprehensive national picture. A further limitation is that contextual determinants such as household income, access to assistive technologies, and availability of local support services were not captured in the 2017 Disability Survey.

Future research should integrate these factors to provide a more comprehensive understanding of the social determinants of communication difficulties. Future research could oversample women and rural dwellers to probe the gender-by-region interaction, incorporate objective language assessments (e.g., picture-naming tasks) into disability surveys, and launch longitudinal cohorts to assess whether new communication-access initiatives under Vision 2030 reduce incidence and severity over time. Mixed-methods studies would also clarify how gender segregation and intercultural pedagogies shape everyday communication experiences in schools and clinics. Additionally, future surveys should complement self-reports with brief, objective language assessments (e.g., picture-naming tasks or comprehension probes) to enhance accuracy and reduce reporting bias.

## 5. Conclusions

Our findings paint a nuanced, data-driven picture: while communication difficulties affect a small proportion of Saudi citizens, the burden is regionally clustered, entwined with chronic disease and consanguinity, and rarely occurs in isolation. Addressing this burden will require an integrated policy mix—accessible content, supportive environments, skilled personnel, and robust monitoring—tailored to the linguistic, cultural, and technological realities. The present study provides an epidemiological baseline against which future interventions can be evaluated, thereby advancing both national disability policy and the global evidence base on communication disorders.

## Figures and Tables

**Table 1:** Total Number of Individuals with Communication Difficulty by Severity and Region.

Region	Mild (n, %)	Severe (n, %)	Extreme (n, %)	Total (n, %)	95% CI for Prevalence
Al-Riyadh	33,712 (26.9%)	10,115 (17.9%)	10,390 (23.3%)	54,217 (23.9%)	1.07–1.11%
Makkah Al-Mokarramah	26,858 (21.4%)	12,046 (21.3%)	14,114 (31.7%)	53,018 (23.4%)	1.04–1.08%
Aseer	15,527 (12.4%)	7537 (13.3%)	4120 (9.2%)	27,184 (12.0%)	1.51–1.60%
Eastern Region	13,658 (10.9%)	6109 (10.8%)	5710 (12.8%)	25,477 (11.2%)	1.08–1.14%
Al-Madinah Al-Monawarah	8104 (6.5%)	6364 (11.3%)	1839 (4.1%)	16,307 (7.2%)	0.70–0.75%
Tabouk	4069 (3.2%)	1900 (3.4%)	1345 (3.0%)	7314 (3.2%)	0.32–0.38%
Hail	4177 (3.3%)	1262 (2.2%)	2006 (4.5%)	7445 (3.3%)	1.44–1.54%
Northern Borders	1395 (1.1%)	810 (1.4%)	559 (1.3%)	2764 (1.2%)	0.18–0.22%
Jazan	9392 (7.5%)	5104 (9.0%)	1440 (3.2%)	15,936 (7.0%)	1.28–1.36%
Najran	846 (0.7%)	621 (1.1%)	508 (1.1%)	1975 (0.9%)	0.42–0.48%
Al-Baha	3051 (2.4%)	1153 (2.0%)	536 (1.2%)	4740 (2.1%)	0.20–0.23%
Al-Jouf	1757 (1.4%)	1159 (2.0%)	241 (0.5%)	3157 (1.4%)	0.14–0.16%
Al-Qaseem	2875 (2.3%)	2329 (4.1%)	1772 (4.0%)	6976 (3.1%)	0.30–0.34%
Total	125,421 (100%)	56,509 (100%)	44,580 (100%)	226,510 (100%)	—

*Note*. Percentages represent the proportion of all Saudi citizens with communication difficulty by region. Approximate 95% confidence intervals are shown in the final column, as PSU/strata variables were not available in GAStat’s public-use files. The narrow intervals reflect the large national sample size, which ensures stable estimates; nonetheless, regional differences remain statistically significant given the limited overlap between high- and low-prevalence areas.

**Table 2:** Gender Distribution of Communication Difficulty by Region.

Region	Male	Female	Total
Al-Riyadh	30,405 (26.1%)	23,812 (21.6%)	54,217 (23.9%)
Makkah Al-Mokarramah	23,851 (22.5%)	29,167 (26.5%)	53,018 (23.4%)
Aseer	14,644 (18.5%)	12,540 (15.9%)	27,184 (12.0%)
Eastern Region	10,529 (14.1%)	14,948 (20.0%)	25,477 (11.2%)
Al-Madinah Al-Monawarah	9859 (14.9%)	6448 (9.7%)	16,307 (7.2%)
Tabouk	4061 (11.7%)	3253 (9.4%)	7314 (3.2%)
Hail	3995 (11.8%)	3450 (10.2%)	7445 (3.3%)
Northern Borders	1624 (10.6%)	1140 (7.4%)	2764 (1.2%)
Jazan	8393 (16.5%)	7543 (14.8%)	15,936 (7.0%)
Najran	1390 (11.1%)	585 (4.7%)	1975 (0.9%)
Al-Baha	2220 (11.2%)	2520 (12.7%)	4740 (2.1%)
Al-Jouf	1363 (10.6%)	1794 (14.0%)	3157 (1.4%)
Al-Qaseem	4605 (20.6%)	2371 (10.6%)	6976 (3.1%)
Total	116,493 (100%)	110,017 (100%)	226,510 (100%)

**Table 3:** Severity Distribution of Communication Difficulty by Gender.

Severity Level	Total	Male	Female
Mild	125,421 (55.4%)	65,928 (56.6%)	59,493 (54.1%)
Severe	56,509 (24.9%)	29,742 (25.5%)	26,767 (24.3%)
Extreme	44,580 (19.7%)	20,823 (17.9%)	23,757 (21.6%)
Total	226,510 (100%)	116,493 (100%)	110,017 (100%)

**Table 4:** Educational Status of Individuals with Communication Difficulty by Gender.

Educational Status	Total	Male	Female
Illiterate	3887 (10.4%)	1543 (9.1%)	2344 (11.5%)
Read and Write	3133 (8.4%)	1316 (7.7%)	1817 (8.9%)
Primary	6952 (18.6%)	3940 (23.2%)	3012 (14.7%)
Intermediate	6307 (16.9%)	2350 (13.8%)	3957 (19.4%)
Secondary/Equivalent	9558 (25.5%)	4577 (26.9%)	4981 (24.4%)
Pre-University Diploma	1913 (5.1%)	1421 (8.3%)	492 (2.4%)
University and Higher	5673 (15.2%)	1851 (10.9%)	3822 (18.7%)
Total	37,423 (100%)	16,998 (100%)	20,425 (100%)

**Table 5:** Marital Status of Individuals with Communication Difficulty by Gender.

Marital Status	Total	Male	Female
Never Married	15,494 (47.0%)	7008 (48.0%)	8486 (46.1%)
Married	16,210 (49.1%)	7099 (48.6%)	9111 (49.5%)
Divorced	1258 (3.8%)	508 (3.5%)	750 (4.1%)
Widowed	77 (0.2%)	0 (0.0%)	77 (0.4%)
Total	33,039 (100%)	14,615 (100%)	18,424 (100%)

**Table 6:** Consanguinity and Communication Difficulty by Gender.

Relationship Between Parents	Total	Male	Female
First degree relatives (father’s side)	6048 (14.7%)	2561 (12.9%)	3487 (16.4%)
First degree relatives (mother’s side)	1324 (3.2%)	794 (4.0%)	530 (2.5%)
First degree relatives (both sides)	8175 (19.9%)	5857 (29.6%)	2318 (11.0%)
Other relatives	4487 (10.9%)	1419 (7.2%)	3068 (14.5%)
Not related	20,968 (51.2%)	9176 (46.3%)	11,792 (55.6%)
Total	41,002 (100%)	19,807 (100%)	21,195 (100%)

**Table 7:** Cause of Disability and Communication Difficulty by Gender.

Cause of Disability	Total	Male	Female
Congenital	10,604 (25.9%)	5980 (30.2%)	4624 (21.9%)
During Pregnancy	850 (2.1%)	85 (0.4%)	765 (3.6%)
During Delivery	3373 (8.2%)	2594 (13.1%)	779 (3.7%)
Traffic Accident	1394 (3.4%)	929 (4.7%)	465 (2.2%)
Other Accident	3631 (8.9%)	2010 (10.1%)	1621 (7.7%)
Disease	14,711 (35.9%)	4969 (25.1%)	9742 (46.1%)
Other	6439 (15.7%)	3240 (16.4%)	3199 (15.1%)
Total	41,002 (100%)	19,807 (100%)	21,195 (100%)

**Table 8:** Duration of Disability and Communication Difficulty by Gender.

Duration of Disability	Total	Male	Female
0–4 years	260 (0.6%)	80 (0.4%)	180 (0.9%)
5–9 years	3319 (8.1%)	2729 (13.8%)	590 (2.8%)
10–14 years	4384 (10.7%)	2383 (12.0%)	2001 (9.5%)
15–19 years	4192 (10.2%)	962 (4.9%)	3230 (15.3%)
20–24 years	4873 (11.9%)	2928 (14.8%)	1945 (9.2%)
25+ years	23,974 (58.5%)	10,725 (54.1%)	13,249 (62.8%)
Total	41,002 (100%)	19,807 (100%)	21,195 (100%)

**Table 9:** Multiple Disabilities and Communication Difficulty by Gender.

Region	Mild	Severe	Extreme	Total
Al-Riyadh	24,686 (25.0%)	8894 (18.3%)	9260 (24.3%)	42,840 (23.1%)
Makkah Al-Mokarramah	20,897 (22.2%)	9806 (20.2%)	12,698 (33.3%)	43,401 (23.4%)
Aseer	12,406 (12.6%)	6776 (13.9%)	3707 (9.7%)	22,889 (12.3%)
Eastern Region	11,447 (11.6%)	5405 (11.1%)	3194 (8.4%)	20,046 (10.8%)
Al-Madinah Al-Monawarah	5893 (6.0%)	5614 (11.5%)	1839 (4.8%)	13,346 (7.2%)
Tabouk	3414 (3.5%)	1337 (2.7%)	942 (2.5%)	5693 (3.1%)
Hail	3270 (3.3%)	860 (1.8%)	1723 (4.5%)	5853 (3.2%)
Northern Borders	1178 (1.2%)	767 (1.6%)	526 (1.4%)	2471 (1.3%)
Jazan	7737 (7.8%)	4708 (9.7%)	1440 (3.8%)	13,885 (7.5%)
Najran	761 (0.8%)	621 (1.3%)	332 (0.9%)	1714 (0.9%)
Al-Baha	2757 (2.8%)	1076 (2.2%)	409 (1.1%)	4242 (2.3%)
Al-Jouf	1408 (1.4%)	1042 (2.1%)	241 (0.6%)	2691 (1.5%)
Al-Qaseem	2875 (2.9%)	1790 (3.7%)	1772 (4.7%)	6437 (3.5%)
Total	98,729 (100%)	48,696 (100%)	38,083 (100%)	185,508 (100%)

**Table 10:** Communication Difficulty by Additional Characteristics (Sign Language Use, Consanguinity, Cause, and Duration, Saudi Arabia 2017).

Variable	Category	Single Disability n (%)	Multiple Disabilities n (%)	Total n (%)
Sign Language Use	Male	–	–	14,150 (51.0)
	Female	–	–	13,598 (49.0)
	Total	–	–	27,748 (100)
Consanguinity	Father’s side	6048 (14.7)	31,866 (17.2)	37,914
	Mother’s side	1324 (3.2)	8970 (4.8)	10,294
	Both sides	8175 (19.9)	40,054 (21.6)	48,229
	Other relatives	4487 (10.9)	33,959 (18.3)	38,446
	Not related	20,968 (51.2)	70,659 (38.1)	91,627
Cause of Disability	Congenital	10,604 (25.9)	40,057 (21.6)	50,661
	Pregnancy	850 (2.1)	7273 (3.9)	8123
	Delivery	3373 (8.2)	24,744 (13.3)	28,117
	Traffic Accident	1394 (3.4)	4643 (2.5)	6037
	Other Accident	3631 (8.9)	10,039 (5.4)	13,670
	Disease	14,711 (35.9)	81,672 (44.0)	96,383
	Other	6439 (15.7)	17,080 (9.2)	23,519
Duration of Disability	0–4 years	260 (0.6)	6917 (3.7)	7177
	5–9 years	3319 (8.1)	14,571 (7.9)	17,890
	10–14 years	4384 (10.7)	11,141 (6.0)	15,525
	15–19 years	4192 (10.2)	10,719 (5.8)	14,911
	20–24 years	4873 (11.9)	17,398 (9.4)	22,271
	25+ years	23,974 (58.5)	124,762 (67.3)	148,736

**Table 11:** Multivariable logistic regression analysis of factors associated with communication difficulty in Saudi Arabia (2017 Disability Survey).

Variable	Category	OR (95% CI)	*p*-Value
Gender	Female vs. Male	0.92 (0.89–0.95)	<0.001
Education Level	University vs. Illiterate	1.46 (1.38–1.55)	<0.001
	Secondary vs. Illiterate	1.17 (1.10–1.24)	<0.001
	Intermediate vs. Illiterate	1.08 (1.02–1.14)	0.011
	Primary vs. Illiterate	1.05 (1.00–1.11)	0.058
	Read and Write vs. Illiterate	0.98 (0.93–1.03)	0.435
Marital Status	Married vs. Never Married	1.05 (1.01–1.09)	0.021
	Divorced vs. Never Married	0.96 (0.90–1.03)	0.264
	Widowed vs. Never Married	0.78 (0.65–0.94)	0.010
Consanguinity	First-degree relatives (both) vs. Not related	1.22 (1.17–1.27)	<0.001
	First-degree relatives (father’s side) vs. Not related	1.13 (1.09–1.17)	<0.001
	First-degree relatives (mother’s side) vs. Not related	1.08 (1.03–1.13)	0.002
	Other relatives vs. Not related	1.18 (1.14–1.22)	<0.001
Cause of Disability	Disease vs. Congenital	1.87 (1.79–1.95)	<0.001
	During Delivery vs. Congenital	2.34 (2.22–2.47)	<0.001
	Other Accident vs. Congenital	0.95 (0.90–1.01)	0.082
	Traffic Accident vs. Congenital	0.44 (0.41–0.47)	<0.001
	During Pregnancy vs. Congenital	0.08 (0.07–0.09)	<0.001
	Other vs. Congenital	0.61 (0.58–0.64)	<0.001
Duration of Disability	25+ years vs. 0–4 years	4.61 (4.25–4.99)	<0.001
	20–24 years vs. 0–4 years	3.22 (2.94–3.53)	<0.001
	15–19 years vs. 0–4 years	2.59 (2.37–2.83)	<0.001
	10–14 years vs. 0–4 years	2.11 (1.93–2.31)	<0.001
	5–9 years vs. 0–4 years	1.84 (1.67–2.02)	<0.001
Use of Sign Language	Yes vs. No	0.88 (0.83–0.93)	<0.001

*Note*. Odds ratios are presented relative to the following reference groups: male, illiterate, never married, not related, congenital cause, disability duration 0–4 years, and no sign language use.

## Data Availability

The data that support the findings of this study are available in General Authority for Statistics, Saudi Arabia at https://www.stats.gov.sa/en/home. (accessed on 1 March 2025) These data were derived from the following resources available in the public domain:—Social Statistics, https://www.stats.gov.sa/en/statistics?index=119025 (accessed on 1 March 2025).

## Supplementary Materials

The following supporting information can be downloaded at: https://www.mdpi.com/article/10.3390/healthcare13192514/s1.
